# Diagnostic Role of 18F-Fluorodeoxyglucose Positron Emission Tomography in Gastric Mesenchymal Tumors

**DOI:** 10.3390/jcm9051301

**Published:** 2020-05-01

**Authors:** Masaya Iwamuro, Koji Miyahara, Chihiro Sakaguchi, Ryuta Takenaka, Sayo Kobayashi, Hirokazu Mouri, Shigetomi Tanaka, Tatsuya Toyokawa, Shouichi Tanaka, Mamoru Nishimura, Kenji Yamauchi, Takehiro Tanaka, Hiroyuki Okada

**Affiliations:** 1 Department of Gastroenterology and Hepatology, Okayama University Graduate School of Medicine, Dentistry and Pharmaceutical Sciences, Okayama 700-8558, Japan; hiro@md.okayama-u.ac.jp; 2 Department of Internal Medicine, Hiroshima City Hospital, Hiroshima 730-8518, Japan; mkojisup@yahoo.co.jp; 3 Department of Endoscopy, National Hospital Organization Shikoku Cancer Center, Matsuyama 791-0280, Japan; chrskgc@yahoo.co.jp; 4 Department of Internal Medicine, Tsuyama Chuo Hospital, Tsuyama 708-0841, Japan; rtakenak@gmail.com; 5 Department of Internal Medicine, Fukuyama City Hospital, Fukuyama 721-8511, Japan; sayo44@hotmail.co.jp; 6 Department of Gastroenterology and Hepatology, Kurashiki Central Hospital, Okayama 710-8602, Japan; hm7882@kchnet.or.jp; 7 Department of Gastroenterology, Kagawa Prefectural Central Hospital, Takamatsu 760-8557, Japan; s-tanaka@chp-kagawa.jp; 8 Department of Gastroenterology, Fukuyama Medical Center, Fukuyama 720-8520, Japan; toyotatu@kmail.plala.or.jp; 9 Department of Gastroenterology, Iwakuni Clinical Center, Iwakuni, Yamaguchi 740-8510, Japan; tanaka.shoichi.pm@mail.hosp.go.jp; 10 Department of Internal Medicine, Okayama City Hospital, Okayama 700-0962, Japan; mamoru_nishimura@okayama-gmc.or.jp; 11 Department of Gastroenterology, Mitoyo General Hospital, Kan-onji 769-1695, Japan; yamauchi.kenji@hotmail.com; 12 Department of Pathology, Okayama University Graduate School of Medicine, Dentistry, and Pharmaceutical Sciences, Okayama 700-8558, Japan; takehiro@md.okayama-u.ac.jp

**Keywords:** 18F-fluorodeoxyglucose-positron emission tomography, mesenchymal tumor, gastric neoplasms, gastrointestinal stromal tumor, schwannoma

## Abstract

There have been no comparative studies investigating the results of 18F-fluorodeoxyglucose (FDG)-positron emission tomography (PET) in patients with gastric mesenchymal tumors, including leiomyomas, leiomyosarcomas, schwannomas, and gastrointestinal stromal tumors (GISTs). We retrospectively reviewed the data of 142 patients with pathologically diagnosed gastric mesenchymal tumors treated at 11 institutions. We analyzed the correlation between the maximum standardized uptake value (SUVmax) evaluated using fluorodeoxyglucose-positron emission tomography (FDG-PET) and the tumor size. The correlation between the SUVmax and mitotic index was also investigated in GISTs. The SUVmax (mean ± standard deviation) was 0.5 ± 0.6 in very low-risk GISTs (*n* = 42), 2.1 ± 0.7 in low-risk GISTs (*n* = 26), 4.9 ± 0.8 in intermediate-risk GISTs (*n* = 22), 12.3 ± 0.8 in high-risk GISTs (*n* = 20), 1.0 ± 1.0 in leiomyomas (*n* = 15), 6.9 ± 1.2 in schwannomas (*n* = 10), and 3.5 in a leiomyosarcoma (*n* = 1). The SUVmax of GISTs with an undetermined risk classification was 4.2 ± 1.3 (*n* = 8). Linear associations were observed between the SUVmax and tumor size in GISTs, leiomyomas, and schwannomas. The SUVmax of GISTs with a high mitotic index was significantly higher than that of GISTs with a low mitotic index (9.6 ± 7.6 vs. 2.4 ± 4.2). In conclusion, we observed positive correlations between the SUVmax and tumor size in GISTs, leiomyomas, and schwannomas. The SUVmax also positively correlated with the mitotic index and risk grade in GISTs. Schwannomas showed a higher FDG uptake than GISTs and leiomyomas.

## 1. Introduction

Gastric mesenchymal tumors include leiomyomas, leiomyosarcomas, schwannomas, and gastrointestinal stromal tumors (GISTs) [1]. Most of these mesenchymal tumors appear as subepithelial tumors, and the surface of the tumor is usually covered with intact gastric mucosa. As a pathological diagnosis with a conventional endoscopic biopsy is generally difficult, imaging studies such as computed tomography, endoscopic ultrasonography, and positron emission tomography (PET) are important for differentiating mesenchymal tumors. PET, a functional imaging technique in nuclear medicine, has been widely used to detect neoplasms in the body. Among the various tracers used in the clinical setting, 18F-fluorodeoxyglucose (FDG) is a representative molecule for PET. As FDG is an analogue of glucose, the tracer concentration corresponds to the regional glucose uptake, thus reflecting tissue metabolic activity. Therefore, uptake of FDG by the tumor is considered to reflect cellular proliferation, and is used as a marker for determining the malignant potential of various neoplasms. In fact, previous studies have demonstrated that the FDG uptake has a significant correlation with the malignant potential of gastric GISTs [2,3]. Meanwhile, gastric schwannomas show an unexpectedly high accumulation of FDG. There have been previous cases of gastric schwannomas misdiagnosed as malignant GISTs based on fluorodeoxyglucose-positron emission tomography (FDG-PET) imaging findings [4]. However, there have been few reports on such cases. In addition, to our knowledge, there have been no comparative studies on the intensity of tracer uptake of different kinds of gastric mesenchymal tumors in FDG-PET. In this study, the FDG-PET results and clinical characteristics of 142 patients with gastric mesenchymal tumors, including 118 GISTs, 15 leiomyomas, 10 schwannomas, and 1 leiomyosarcoma, were retrospectively examined. The aim of the current study was to investigate the role of FDG-PET and the findings that need cautious interpretation in the preoperative diagnosis of gastric mesenchymal tumors.

## 2. Methods

Letters of inquiry to patients with gastric mesenchymal tumors were sent from the Department of Gastroenterology and Hepatology, Okayama University Graduate School of Medicine, Dentistry, and Pharmaceutical Sciences to 10 collaborating institutions. The inclusion criteria were (1) having pathologically diagnosed gastric mesenchymal tumors, including leiomyomas, leiomyosarcomas, schwannomas, and GISTs, and (2) having undergone FDG-PET for gastric mesenchymal tumors. Pathological diagnosis was made on the basis of the findings of endoscopic biopsy, endoscopic ultrasound-fine needle aspiration biopsy, endoscopic submucosal dissection, and/or surgical resection. We identified 142 patients who had been diagnosed with 144 gastric mesenchymal tumors and underwent FDG-PET between December 2006 and December 2018. These patients were retrospectively registered in this study.

We retrospectively examined the patients’ sex, age at diagnosis, complications, modalities undergone for pathological diagnosis, maximum standardized uptake value (SUVmax) evaluated using FDG-PET, tumor size, and prognosis. The follow-up period was defined as the time from the diagnosis of gastric mesenchymal tumors to death of any cause, or the last hospital visit. Pathological features such as the mitotic index and the presence or absence of tumor rupture were also investigated in GISTs. The risk of GISTs was classified according to the modified National Institutes of Health classification suggested by Joensuu [5]. Briefly, the mitotic index was quantified by counting the number of mitotic cells per 50 high-power fields (HPFs) on hematoxylin and eosin staining. Gastric GISTs with a diameter of ≤2 cm and a mitotic index of ≤5 were defined as “very low risk.” Gastric GISTs 2.1–5.0 cm in diameter and with a mitotic index of ≤5 were classified as “low risk.” Gastric GISTs ≤ 5.0 cm in diameter and with a mitotic index of 6–10 were defined as “intermediate risk.” Gastric GISTs 5.1–10.0 cm in diameter and with a mitotic index of ≤5 were also classified as “intermediate risk.” Other gastric GISTs were classified as “high risk.” Gastric GISTs with ruptures were defined as “high risk,” irrespective of the tumor size and mitotic index. The primary endpoint of this study was the correlation of the SUVmax and the pathological types of gastric mesenchymal tumors.

Statistical analyses were performed using JMP 14.0.0 software (SAS Institute Inc., Cary, NC, USA) and a one-way analysis of variance followed by a Tukey–Kramer post hoc test for multiple comparisons. *P* < 0.05 was considered to indicate a statistically significant difference. The present study was approved by the Ethics Committees of Okayama University Hospital and other institutions, and adhered to the Declaration of Helsinki.

## 3. Results

The characteristics of the enrolled patients are shown in Table 1. There were 90 men and 52 women. The mean age at the diagnosis of gastric mesenchymal tumors was 68.2 years (range: 15–89 years). Pathological diagnosis was made on the basis of surgically resected specimens (n = 103), endoscopic ultrasound-guided fine-needle aspiration biopsy specimens (n = 31), endoscopic forceps biopsy specimens (n = 9), and endoscopic submucosal dissection specimen (n = 1). The pathological diagnoses were GIST (n = 118), leiomyoma (n = 15), schwannoma (n = 10), and leiomyosarcoma (n = 1). The risk grades of GISTs according to the modified National Institutes of Health classification were very low risk (n = 42), low risk (n = 26), intermediate risk (n = 22), and high risk (n = 20). The risk of GISTs was not categorized in five patients because the mitotic index was not evaluated, owing to the small sample size from endoscopic ultrasound-guided fine-needle aspiration biopsy. Risk classification was also unavailable in the other three patients who underwent surgical resection of GISTs because the mitotic index was not pathologically evaluated. Consequently, the risk of GISTs was unclassified in eight patients. Among the 142 patients, 1 patient had both a high-risk GIST and an intermediate-risk GIST. Another patient had one very low-risk GIST and one leiomyoma. The mean size of the gastric mesenchymal tumors was 3.7 cm (range: 0.1–23.0 cm). The mean size of GISTs was 3.9 cm, that of leiomyomas was 1.4 cm, and that of schwannomas was 5.7 cm. The size of the leiomyosarcoma was 4.2 cm. One-way analysis of variance followed by a Tukey–Kramer post hoc test found no differences in tumor sizes among the pathological types.

**Figure 1:**
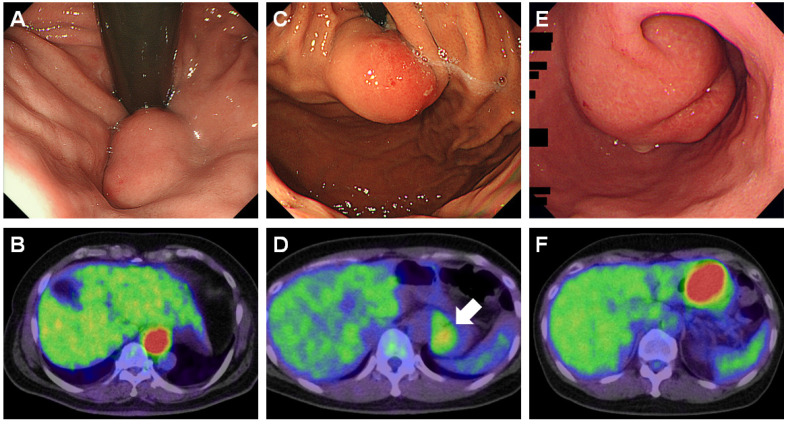
Representative endoscopic and 18F-fluorodeoxyglucose (FDG)-positron emission tomography images of gastric gastrointestinal stromal tumor (GIST), leiomyoma, and schwannoma. High-grade GIST that presented as a subepithelial tumor in the gastric cardia (**A**) and showed increased FDG uptake (**B**, maximum standardized uptake value [SUVmax] = 11.4, tumor size: 4.6 cm). A gastric leiomyoma was observed as a subepithelial lesion in the cardia (**C**). The SUVmax of the leiomyoma was 2.75 (**D**, tumor size: 2.1 cm). A schwannoma in the gastric body (**E**) showed increased tracer accumulation (**F**, SUVmax = 9.01, tumor size: 5.5 cm).

Figure 1 shows representative endoscopic and FDG-PET images of a gastric GIST (Figure 1A,B), leiomyoma (Figure 1C,D), and schwannoma (Figure 1E,F). FDG-PET was performed for a detailed examination of gastric subepithelial lesions in 75 patients and for the examination of other diseases in 65 patients, including esophageal cancer (*n* = 19), gastric cancer (*n* = 15), lung cancer (*n* = 9), colorectal cancer (*n* = 6), breast cancer (*n* = 5), pancreatic cancer (*n* = 3), laryngeal cancer (*n* = 2), gallbladder cancer (*n* = 2), extranodal marginal zone lymphoma of mucosa-associated lymphoid tissue (MALT lymphoma) in the stomach (*n* = 1), uterus cancer (*n* = 1), prostate cancer (*n* = 1), liver cancer (*n* = 1), peritoneal pseudomyxoma (*n* = 1), lymphadenopathies (*n* = 1), and peritoneal tumors (*n* = 1). Among them, three patients had double primary cancers: two patients had esophageal and gastric cancers and the other patient had gastric and gallbladder cancers. Two patients underwent FDG-PET for cancer screening without any symptoms or underlying diseases.

**Figure 2:**
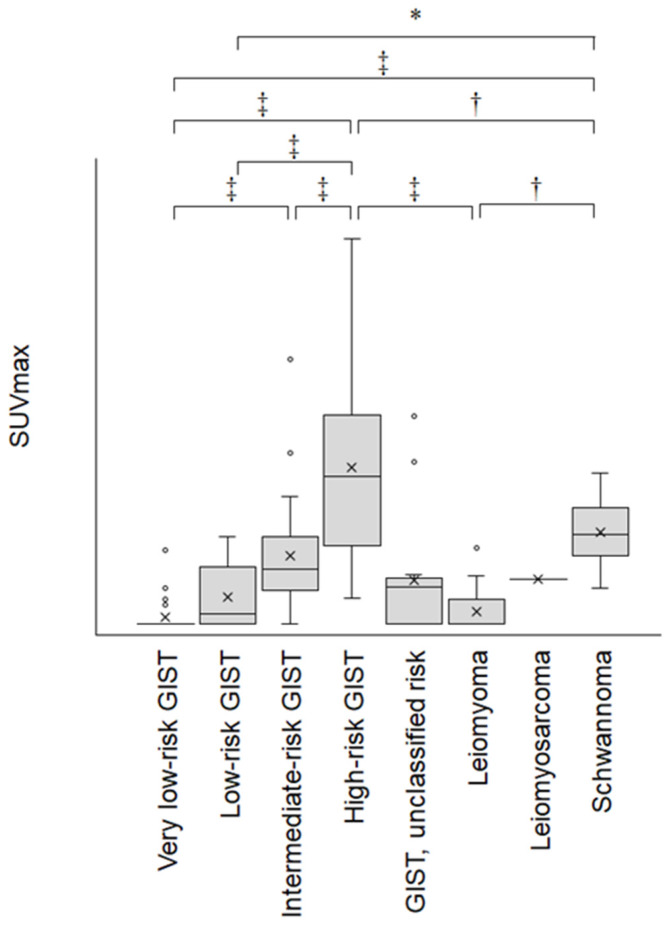
Box-and-whisker plots of the SUVmax of gastric mesenchymal tumors. * *P* < 0.05; † *P* < 0.01; ‡ *P* < 0.001.

The SUVmax of mesenchymal tumors are shown in Figure 2. The mean ± standard deviation (SD) of the SUVmax was 0.5 ± 0.6 in very low-risk GISTs, 2.1 ± 0.7 in low-risk GISTs, 4.9 ± 0.8 in intermediate-risk GISTs, 12.3 ± 0.8 in high-risk GISTs, 1.0 ± 1.0 in leiomyomas, and 6.9 ± 1.2 in schwannomas. The SUVmax of the leiomyosarcoma was 3.5. The SUVmax of GISTs with an undetermined risk classification was 4.2 ± 1.3. With respect to the SUVmax in each pathological type, one-way analysis of variance followed by a Tukey–Kramer post hoc test found significant differences in several types (Table 2, Figure 2).

**Figure 3:**
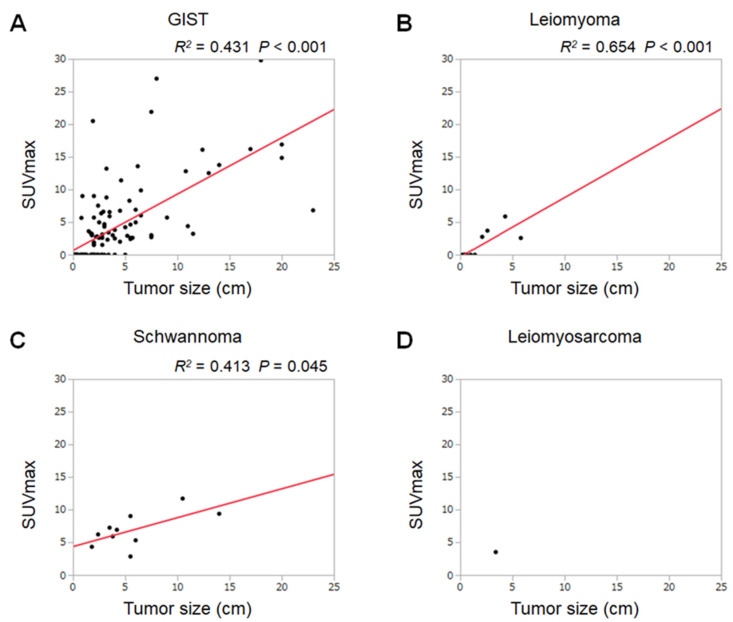
Scatter diagrams and regression lines with respect to tumor sizes and the SUVmax of GISTs (**A**), leiomyomas (**B**), schwannomas (**C**), and leiomyosarcoma (**D**).

Figure 3 shows scatterplots based on the SUVmax and tumor sizes in each pathological type. The *R*^2^ values were 0.431 (*p* < 0.001) in GISTs, 0.654 (*p* < 0.001) in leiomyomas, and 0.413 (*p* = 0.045) in schwannomas, indicating that the SUVmax correlated with the tumor sizes in these pathological types. On the basis of linear regression analysis of the scatterplots, the SUVmax of GISTs was estimated as 0.66 + 0.86 × tumor size (cm), that of leiomyomas as −0.30 + 0.91 × tumor size (cm), and that of schwannomas as 4.34 + 0.44 × tumor size (cm).

**Figure 4:**
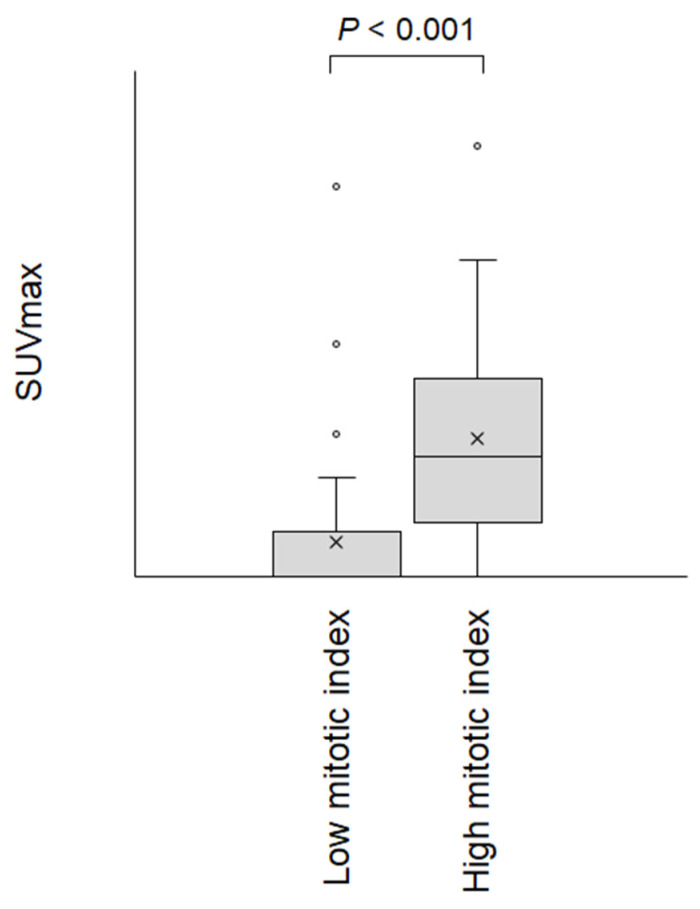
Dot-plot diagram showing the SUVmax of gastrointestinal stromal tumors with a low mitotic index (≤5 mitoses/50 high-power fields) and a high mitotic index (≥6 mitoses/50 high-power fields).

**Figure 5:**
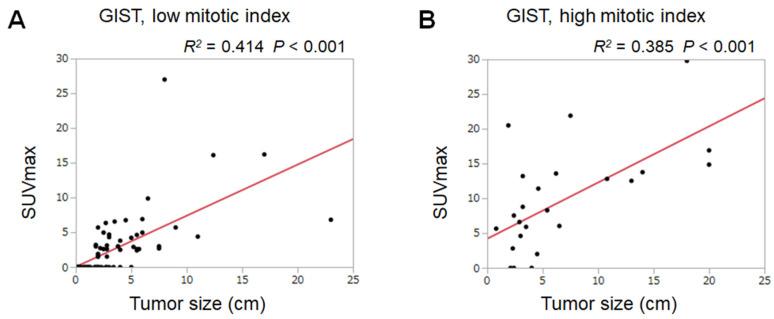
Scatter diagrams and regression lines with respect to tumor sizes and the SUVmax of gastrointestinal stromal tumors with a low mitotic index (≤5 mitoses/50 high-power fields, **A**) and a high mitotic index (≥6 mitoses/50 high-power fields, **B**).

Box plots of GISTs with a low mitotic index (≤5 mitoses/50 high-power fields) and GISTs with a high mitotic index (≥6 mitoses/50 high-power fields) are displayed in Figure 4. The SUVmax of GISTs with a high mitotic index (mean ± SD: 9.6 ± 7.6) was significantly higher than that of GISTs with a low mitotic index (2.4 ± 4.2), indicating that the SUVmax reflects the cell proliferation of GISTs. To reveal correlations between SUVmax, tumor size, and mitotic index, we performed linear regression analysis in the low and high mitotic index groups (Figure 5). The *R*^2^ values were 0.414 in GISTs with a low mitotic index and 0.385 in GISTs with a high mitotic index. The SUVmax of GISTs with a low mitotic index was estimated as 0.05 + 0.73 × tumor size (cm), whereas that of GISTs with a high mitotic index was estimated as 4.20 + 0.81 × tumor size (cm).

The mean follow-up period after the pathological diagnosis of gastric mesenchymal tumors was 3.6 years (range: 0.0–11.3 years). Among the 142 patients, 114 patients were alive at the last visit to each institution, whereas 24 patients died of causes other than gastric mesenchymal tumors. The remaining four patients died owing to the progression of GISTs. The SUVmax of GISTs in these four patients was 2,0, 13.8, 14.9, and 16.1, respectively. The SUVmax in patients who died because of GIST progression (mean ± SD: 11.7 ± 6.5) was significantly higher than that in the other patients (3.7 ± 5.3) (*p* < 0.01).

## 4. Discussion

To our knowledge, there have been no studies comparing the FDG-PET results among different pathological types of gastric mesenchymal tumors. In addition, our study is the largest to date investigating the FDG avidity among gastric GISTs. We revealed that the SUVmax increases as the risk of GISTs becomes higher (Figure 2). Previous reports have also described a positive correlation between the SUVmax and risk grade [2,6,7,8,9]. A significant correlation between the SUVmax and mitotic index has also been reported [7,8,10,11]. Meanwhile, mixed results have been noted with respect to tumor size, in that several reports failed to show a correlation between FDG avidity and the size of GISTs [8,10]. We consider that this issue was due to the small sample sizes in previous reports. As FDG-PET visualizes the glycolytic activity of a tumor, it is likely that both a large tumor size and a high proliferation potential result in increased FDG uptake [12,13]. Moreover, because the risk grades of GISTs are defined based on the tumor size and mitotic index, a positive correlation between the SUVmax and risk grade seems reasonable. Other factors, such as the Ki-67 percentage score and Glut-1 expression, which correlate with SUVmax in bone and soft tissue sarcomas [14], might also be involved in gastric mesenchymal tumors. However, because we did not perform Ki-67 staining or Glut-1 expression analysis, relationships between these features and SUVmax have not been elucidated.

Linear regression analysis revealed that the SUVmax of GISTs and leiomyomas are mostly in direct proportion to the tumor size (Figure 3). The inclination of the linear estimation is noteworthy: the constant of proportionality of leiomyomas (0.91) is similar to that of GISTs (0.86). These results indicate that, although huge leiomyomas unlikely exist (owing to their benign nature), larger leiomyomas would show a greater increase in FDG uptake, as observed in GISTs. Consequently, differentiation of GISTs from leiomyomas based on FDG-PET results is impossible. In a previous report, a patient with an esophageal leiomyoma misdiagnosed as a GIST preoperatively was described [15]. Since the number of patients with leiomyomas included in this study was relatively small, further investigation is required to elucidate this issue.

Our study revealed increased FDG uptake in schwannomas, which was significantly higher than that in very low-risk GISTs and low-risk GISTs. Since Komatsu et al. first reported a patient with gastric schwannoma that was positive for FDG-PET (SUVmax: 5.8.) [16], there have been several case reports and case series describing such patients. Fujiwara et al. described that all of their four patients with gastric schwannoma showed FDG uptake and the SUVmax ranged from 3.3 to 6.8 (median: 4.7) [17]. Ohno et al. also reported two cases of gastric schwannomas showing an SUVmax of 6.05 and 7.10, respectively [18]. In the present study, the SUVmax of schwannomas was 6.9 ± 1.2 (range: 2.8–11.7). Thus, our study reinforces the notion that all gastric schwannomas have avidity for FDG. Particularly, the value of the y-intercept in the linear regression for schwannomas was 4.34, whereas the SUVmax of GISTs and leiomyomas was almost directly proportional to the tumor sizes. These results indicate that small GISTs and leiomyomas are negative or slightly positive for FDG accumulation, whereas schwannomas, even if small, show increased FDG uptake. Thus, gastroenterologists must take caution not to misinterpret the FDG-PET results of gastric schwannomas as intermediate- or high-risk GISTs.

This study had several limitations. First, 18F-FDG-PET was performed under different conditions, as the included patients had been treated at various institutions. For example, the period between the intravenous administration of FDG and the initiation of FDG-PET varied between 60 and 120 min. It is possible that other differences in methodology among the participating institutions may also have affected the positivity of FDG uptake and the SUVmax [19,20]. Since the number of patients per institution varied from 1 to 36 (median: 11), separating and comparing data among institutions would have no statistical validity. Conversely, our results could probably be generalized to institutions worldwide. Second, the follow-up period after the pathological diagnosis of gastric mesenchymal tumors was relatively short (mean: 3.6 years), resulting in insufficient analysis of the patients’ outcome. Complete follow-up with a longer observation period is desirable to evaluate the correlation between the SUVmax and prognosis, particularly in GISTs of higher risk grades. Third, the sample size of each group was small. As gastric mesenchymal tumors are relatively infrequent, nationwide investigations are desirable to reveal the true nature of FDG avidity in these tumors.

In conclusion, we comparatively investigated the FDG-PET results of 118 GISTs, 15 leiomyomas, 10 schwannomas, and one leiomyosarcoma, and revealed positive correlations between the SUVmax and tumor size in GISTs, leiomyomas, and schwannomas. The SUVmax also positively correlated with the mitotic index and risk grade in GISTs. Schwannomas showed a higher FDG uptake than GISTs and leiomyomas. These results highlight that gastric schwannomas can be misinterpreted as intermediate- or high-risk GISTs on FDG-PET examination. Thus, careful interpretation of the FDG-PET results is required for the preoperative differential diagnosis of gastric mesenchymal tumors.

## Figures and Tables

**Table 1:** Clinical characteristics of the study patients.

	N
Sex	
Male	90
Female	52
Mean age (range), years	68.2 (15–89)
Histology	
GIST	118
Very low risk	42
Low risk	26
Intermediate risk	22
High risk	20
Unclassified *	8
Leiomyoma	15
Schwannoma	10
Leiomyosarcoma	1
Methods for pathological diagnosis	
Surgical resection	103
EUS-FNA	31
Endoscopic forceps biopsy	9
ESD	1
FDG-PET was performed for	
Gastric subepithelial lesions	77
Other diseases	65
Cancer screening	2
Mean tumor size (range), cm	3.7 (0.1–23.0)
Mean follow-up period (range), years	3.6 (0.0–11.3)
Outcome	
Alive	114
Died of GIST	4
Died of other diseases	24

GIST, gastrointestinal stromal tumor; EUS-FNA, endoscopic ultrasound-fine-needle aspiration biopsy; ESD, endoscopic submucosal dissection; FDG-PET, 18F-fluorodeoxyglucose-positron emission tomography. * The risk of GISTs was not categorized because the mitotic index was not evaluated.

**Table 2:** Results of one-way analysis of variance followed by Tukey–Kramer post hoc test with respect to the SUVmax across pathological types.

	*P* Value
High-risk GIST vs. very low-risk GIST	<0.0001
High-risk GIST vs. leiomyoma	<0.0001
High-risk GIST vs. low-risk GIST	<0.0001
High-risk GIST vs. leiomyosarcoma	0.3126
High-risk GIST vs. intermediate-risk GIST	<0.0001
Schwannoma vs. very low-risk GIST	0.0001
Schwannoma vs. leiomyoma	0.0051
High-risk GIST vs. schwannoma	0.0061
Schwannoma vs. low-risk GIST	0.0217
Intermediate-risk GIST vs. very low-risk GIST	0.0007
Intermediate-risk GIST vs. leiomyoma	0.0515
Schwannoma vs. leiomyosarcoma	0.9898
Leiomyosarcoma vs. very low-risk GIST	0.9940
Intermediate-risk GIST vs. low-risk GIST	0.2011
Leiomyosarcoma vs. leiomyoma	0.9982
Schwannoma vs. intermediate-risk GIST	0.8802
Low-risk GIST vs. very low-risk GIST	0.6802
Intermediate-risk GIST vs. leiomyosarcoma	1.0000
Leiomyosarcoma vs. low-risk GIST	1.0000
Low-risk GIST vs. leiomyoma	0.9827
Leiomyoma vs. very low-risk GIST	0.9999

SUVmax, maximum standardized uptake value; GIST, gastrointestinal stromal tumor.
